# Focusing on Mechanoregulation Axis in Fibrosis: Sensing, Transduction and Effecting

**DOI:** 10.3389/fmolb.2022.804680

**Published:** 2022-03-11

**Authors:** Dongsheng Wen, Ya Gao, Chiakang Ho, Li Yu, Yuguang Zhang, Guozhong Lyu, Dahai Hu, Qingfeng Li, Yifan Zhang

**Affiliations:** ^1^ Department of Plastic and Reconstructive Surgery, Shanghai Ninth People’s Hospital, Shanghai Jiao Tong University School of Medicine, Shanghai, China; ^2^ Department of Burns and Plastic Surgery, Affiliated Hospital of Jiangnan University, Wuxi, China; ^3^ Burns Centre of PLA, Department of Burns and Cutaneous Surgery, Xijing Hospital, Fourth Military Medical University, Xi’an, China

**Keywords:** fibrosis, mechanosensing, mechanotransduction, epigenetic modification, clinical trials

## Abstract

Fibrosis, a pathologic process featured by the excessive deposition of connective tissue components, can affect virtually every organ and has no satisfactory therapy yet. Fibrotic diseases are often associated with organ dysfunction which leads to high morbidity and mortality. Biomechanical stmuli and the corresponding cellular response havebeen identified in fibrogenesis, as the fibrotic remodeling could be seen as the incapacity to reestablish mechanical homeostasis: along with extracellular matrix accumulating, the physical property became more “stiff” and could in turn induce fibrosis. In this review, we provide a comprehensive overview of mechanoregulation in fibrosis, from initialing cellular mechanosensing to intracellular mechanotransduction and processing, and ends up in mechanoeffecting. Our contents are not limited to the cellular mechanism, but further expand to the disorders involved and current clinical trials, providing an insight into the disease and hopefully inspiring new approaches for the treatment of tissue fibrosis.

## 1 Introduction

Fibrosis is a process featuring excessive deposition of extracellular matrix (ECM) proteins, which leads to scarring and thickening of the affected tissue (Rockey et al., 2015). With the up-to-date understanding of fibrosis, biomechanics have been recognized in numerous fibroproliferative diseases (Eckes et al., 2000; Tomasek et al., 2002; Gao et al., 2019). Under physiological conditions, cells are constantly exposed to mechanical forces, such as blood pressure and shear stress generated by blood flow, expiratory and inspiratory forces, and compressive or tensile stresses from the skin and musculoskeletal system (Mammoto et al., 2013). Cells can sense changes in the physical environment, and subsequently transduce extracellular mechanical signals into intracellular biochemical reactions and gene expression regulation (Saucerman et al., 2019). When the mechanical homeostasis is disrupted, fibroblast activation becomes uncontrolled and finally results in amplified ECM generation (Tschumperlin et al., 2018). The progressive deposition of ECM results in tissue stiffening, leading to a self-amplifying loop of fibroblast activation, and finally providing a greater mechanical context for fibrogenesis. In addition, mechanical stress regulates cellular mechanosensitive signaling pathways (Provenzano et al., 2009), which influence cell metabolism (Zhao et al., 2020) and remodel the immune microenvironment (Brown and Marshall, 2019), and epithelial-mesenchymal transition (EMT) (Stone et al., 2016). Therefore, understanding how biophysical parameters of the mechanical environment regulate cell behavior is of great importance in fibrosis.

In this review, we summarize the progress of the cell response to physical forces and discuss the cell mechanosensation, mechanotransduction and mechanoeffecting. We predominantly focus on the shared mechanism. This review also expands our vision from the laboratory bench to the clinical bedside, considering the related diseases and the latest clinical trials. We believe that a deeper understanding of biomechanics could provide new insights into mechanoregulation in fibrotic tissue remodeling, and help us identify novel therapies.

## 2 Cellular Mechanosensing in Fibrosis

**FIGURE 1:**
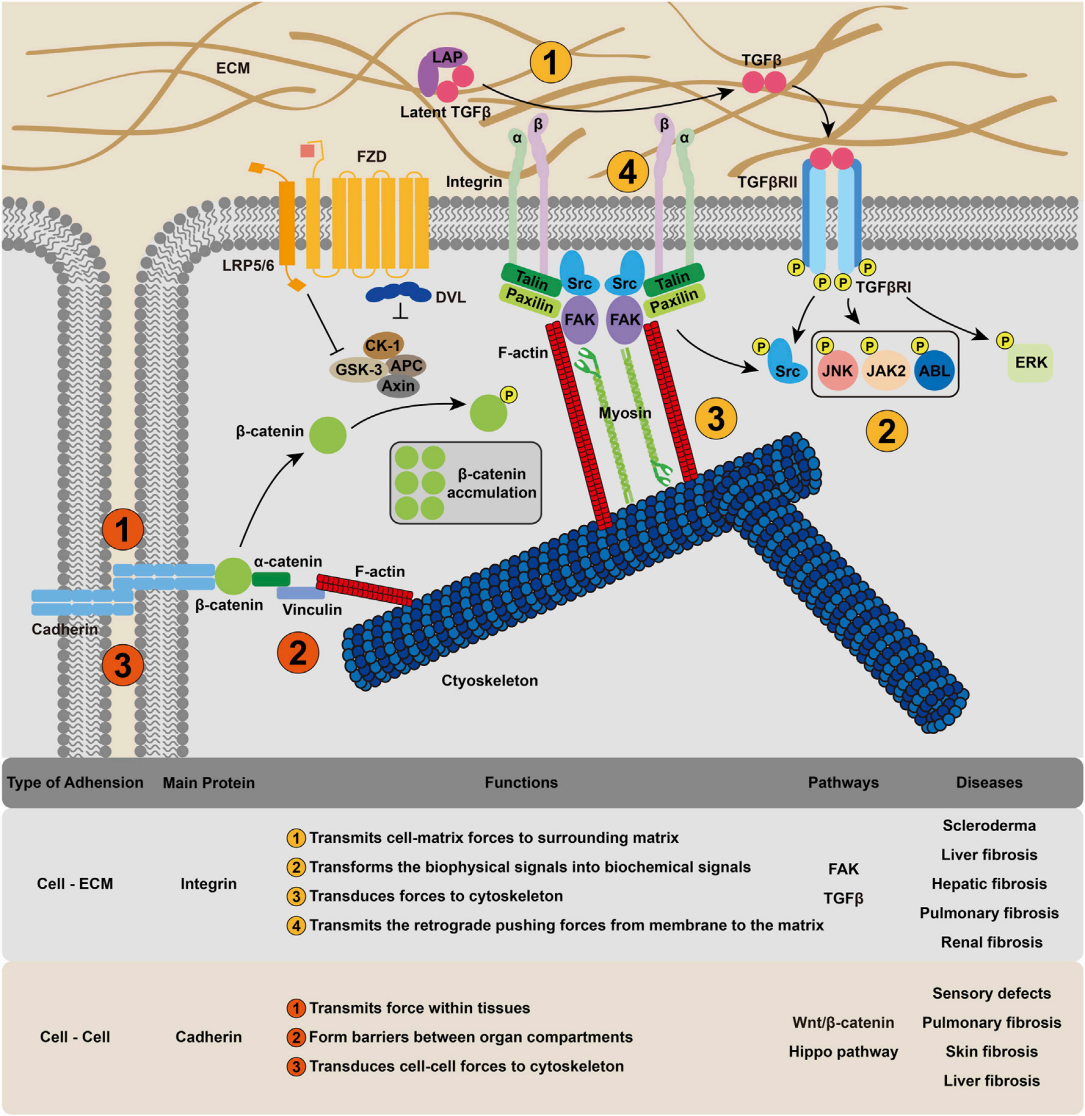
Cellular mechanosensing mechanism in fibrosis. Cell could receive and intergrate mechanical signals through integrin-based cell–ECM interactions and cadherin-based cell–cell interactions, transforming the extracellular physical cues into membrane tension and cytoskeleton deformation.

A number of sensory elements and mechanisms enable cells to detect and react to extracellular forces through a process named mechanosensing. This force-sensing process can occur through force-induced conformational or organizational changes in cellular molecules and structures, including stretch-sensitive ion channels (Wang et al., 2015; Northey et al., 2017), cadherin complexes (Leckband and de Rooij, 2014), G protein-coupled receptors (GPCRs) and integrins (Herrmann et al., 2020). The ion channels on the cell membrane have both mechanosensing and mechanotransduction functions and will be discussed in the next section. In this section, we classify the cellular mechanosensing into cell–matrix and cell–cell mechanosensing (Figure 1), and the detailed mechanism, diseases involved, and recent clinical trials will be discussed.

### 2.1 Mechanisms of Cell-ECM Mechanosensing

Integrins provide a mechanical linkage between the ECM and the cytoskeleton (Humphrey et al., 2014). In addition, integrins can also act as mechanosensors (Papusheva and Heisenberg, 2010) and morphogenetic regulators that modify cell–ECM adhesion (Herrmann et al., 2020). As the name suggests, integrins are cell surface receptors that integrate the cytoskeleton into the extracellular environment. They are composed of noncovalent *α*, *ß* heterodimers. Not all integrins are constitutively active, and their activation starts with conformational changes in the integrin ectodomain (Sun et al., 2016). Kindlin and talin bind integrin cytoplasmic tails to promote integrin activation (Gough and Goult, 2018). Once bound, integrins recruit numerous proteins, such as LIM domains, to their short cytoplasmic tails, resulting in the assembly of various adhesion structures (Schiller and Fässler, 2013). Thus, the molecular clutch, a mechanical linkage composed of dynamic associations between the ECM-bound integrins and the force-generating actomyosin cytoskeleton, is formed (Mitchison and Kirschner, 1988). Sensing the matrix rigidity, cells employ this molecular clutch to transmit forces to their surrounding matrix (Elosegui-Artola et al., 2014) and then transduce biomechanical into biochemical signals (Dupont, 2016). For example, in response to elevated mechanical loading, integrin clustering can recruite and phosphorylate focal adhesion kinase (FAK), and phospho-FAK initiates a cascade of signaling events, such as cell migration and inflammatory signaling (Wong et al., 2011), to induce fibrosis. The adhesions are also able to transmit retrograde pushing forces from the polymerizing branched actin network in membrane protrusions to the ECM via proteins such as talin and vinculin (Hu et al., 2007). Interestingly, integrin is also recognized in the TGFβ signaling pathway, which is one of the most important regulators of fibrosis (Meng et al., 2016). Integrins are highly expressed in activated fibroblasts (Reed et al., 2015) and interact with a linear arginine-glycine-aspartic acid (RGD) motif present in TGF*β* complexes with latency-associated peptide (LAP) to transform TGF*β* to its active form (Distler et al., 2019).

Increased expression of *α*v*β*3 or *α*v*β*5 integrins is observed in the dermis of scleroderma patients (Hinchcliff and O’Reilly, 2020), and these integrins induce autocrine TGF*β* signaling in patient fibroblasts *in vitro* (Santos and Lagares, 2018), suggesting that *α*v*β*3/5-mediated TGFβ activation could be important under pathological conditions (Brown and Marshall, 2019).

### 2.2 Mechanisms of Cell–Cell Mechanosensing

Mechanical forces exerted on cell–cell adhesions that link adjacent cells also play an important role. Intercellular contacts, particularly cadherin-based intercellular junctions, are the major means of transmitting force within tissues (Herrmann et al., 2020). The classical extracellular cadherin domain folds into five *ß*-barrel parts and embeds the primary adhesion site. There is a single-pass transmembrane domain and a cytoplasmic domain, which bind different cytoplasmic and cytoskeletal proteins. For the cytodomain, the main interactors include p120ctn, *ß*-catenin and *a*-catenin, which can bind to F-actin directly or through vinculin (Buckley et al., 2014). The adhesion of cadherin requires the formation of cadherin–cadherin bonds, which include different structural regions and exhibit different kinetic and mechanical properties (Rakshit et al., 2012; Priest et al., 2017). F-actin-associated classical cadherin complexes have been shown to be mechanosensors (Leckband and de Rooij, 2014; Bays and DeMali, 2017; Dasgupta and McCollum, 2019; Hur et al., 2020). *a*-catenin, which links E-cadherin-associated *ß*-catenin to F-actin (Desai et al., 2013), is the central molecule in this system. The best characterized effector of the tension-dependent conformational regulation of *a*-catenin is its closest homolog, vinculin (Leckband and de Rooij, 2014), which can be recruited to cell–cell interactions in response to endogenous myosin II–dependent contractility (Kuehlmann et al., 2020) and externally applied tension (Thomas et al., 2013). Cadherins have also been identified in the transcriptional regulation of the Wnt/*β*-catenin (Sun et al., 2014) and Hippo pathways (Gumbiner and Kim, 2014). Beyond sensing, cell–cell adhesions are critical for the formation of barriers between organs or between the body and the external environment (Hintermann and Christen, 2019).

Defects in cadherin can result in multiple disorders, including skin and hair malfunctions, cardiomyopathies, sensory defects associated with deafness and blindness and psychiatric disorders (El-Amraoui and Petit, 2013). An upregulation of cadherin has been observed in fibrotic lung and skin tissue (Agarwal, 2014; Madarampalli et al., 2019). Recent studies have also confirmed that cadherin-11 contributes to liver fibrosis caused by carbon tetrachloride (Pedroza et al., 2019) and that the level of cadherin-11 correlates with the fibrosis stage (Ruan et al., 2019). In the process of EMT, a cadherin switch, the upregulation of N-cadherin and the downregulation of E-cadherin, has been found (Loh et al., 2019). Taken together, these findings suggest that cadherin could be a potential target for fibrotic treatments. In the FOXF1-deleted mouse model, an increase in the switch from N-cadherin to cadherin-11, which is a critical step in the acquisition of the profibrotic phenotype, was observed (Black et al., 2018), suggesting that FOXF1 inhibited pulmonary fibrosis by regulating the cadherin switch.

## 3 Cellular Mechanotransduction in Fibrosis

**FIGURE 2:**
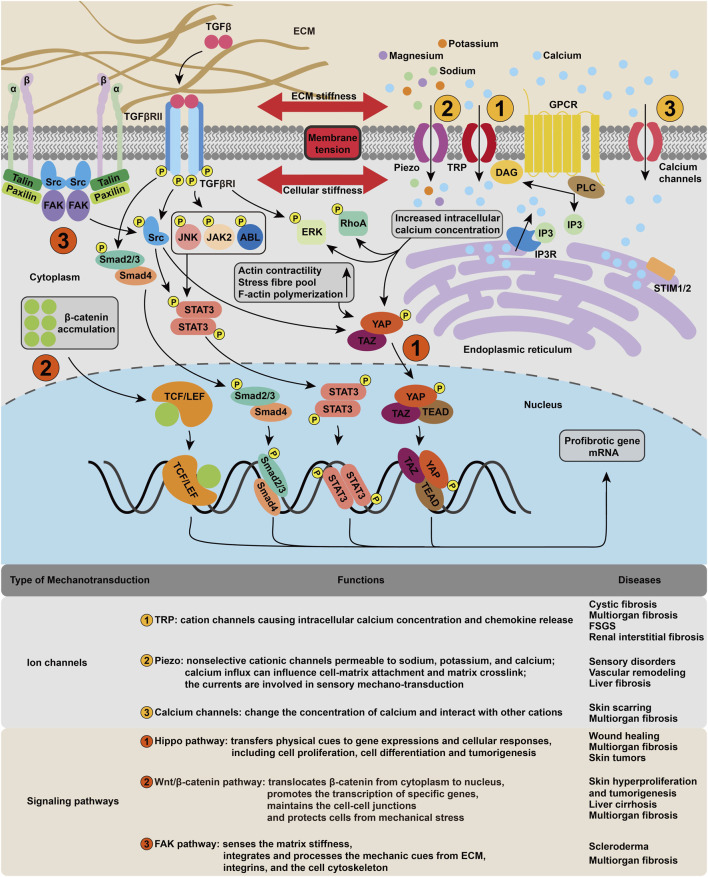
Cellular mechanotransduction mechanism in fibrosis. The membrane tension activates various mechanosensitive ion channels, such as piezo and TRP, resulting in the change of intracellular ion concentration, which in turn leads to the upregulation of signaling pathways including ERK, RhoA. The cytoskeleton deformation regulates fibrotic gene expression mainly through FAK, Hippo and Wnt/*β*-catenin pathways.

Once the mechanical cue passes through the cell membrane, multiple downstream biochemical pathways, including calcium-dependent pathways, nitric oxide (NO) signaling, mitogen-activated protein kinases (MAPK), Rho GTPases, and phosphoinositol-3-kinase (PI3K), are activated (Isermann and Lammerding, 2013). Apart from these signaling pathways, some biomechanical-related transcription factors and coregulators, mechanosensitive ion channels and microRNAs are also involved (Wang et al., 2015). The mechanotransduction process transforms biomechanical into biochemical or electrochemical signals, which can be recognized by intracellular components. In this section, we will focus on the ion channels and signaling pathways (Figure 2).

### 3.1 Ion Channels in Cellular Mechanotransduction

Mechanical stimuli are observed to induce ionic currents through different ion channels in the cell membrane (Del Favero and Kraegeloh, 2020). Channels previously considered voltage-gated, such as potassium and sodium channels, are also found to be mechanically sensitive (Wang et al., 2015). The opening of ion channels in response to pressure stimulation or shear stress is the earliest cellular event that induces cell depolarization (Douguet et al., 2019).

#### 3.1.1 Transient Receptor Potential Channels

The TRP protein superfamily functions as calcium channels and is widely expressed in various organs. There are 28 mammalian TRP subunits, categorized into six related protein subfamilies (TRPC1-7, TRPV1-6, TRPM1-8, TRPA1, TRPML1-3, TRPP1-2) based on sequence homology (Jiao et al., 2017). The universal expression of TRP proteins may explain their active participation in different organs and their involvement in various mechanosensory processes, including blood pressure regulation, touch sensation, and bone loading. TRP channels can be directly mechanically gated or gated through a multistep cascade. The mechanical force exerted on the TRP channels or on the membrane opens the ion channel by switching the conformation of TRPs or by altering the curvature of the lipid bilayer. Similarly, a nonmechanical stimulus, such as light or a chemical agonist, can trigger a signaling cascade on the lipid bilayer and generate forces to mechanically activate TRP channels (Liu and Montell, 2015). This cascade starts with the activation of a GPCR, and then the phospholipase C is activated to hydrolyze the phosphatidylinositol 4,5-bisphosphate and produce the inositol 1,4,5-trisphosphate and diacylglycerol (DAG). The cleavage of the DAG causes a change in the cell membrane and leads to the activation of TRP channels.

TRPM7 is markedly upregulated in atrial fibrillation (AF) patients, activating the calcineurin pathway and producing a synergistic effect with TGFβ1, and resulting in the activation of fibroblasts. These activities are eliminated with usage of the TRPM7 blocker 2-APB or RNA interference specific for TRPM7 (Nattel, 2017). In human AF patients and dog AF models, TRPC3 expression is upregulated with enhanced nonselective cation influxes and increased *a*-smooth muscle actin (α-SMA) expression and extracellular regulated protein kinases 1/2 (ERK 1/2) phosphorylation, which can be suppressed by the TRPC3-selective blocker Pyrazol-3 (Harada et al., 2012; Hall et al., 2019). The expression of TRPC1 and TRPM7 has also been evaluated, which was much higher than that of TRPC3, and remained unaffected under AF-inducing conditions, suggesting that TRPC3-ERK signaling is of greater significance in fibrogenesis (Inoue et al., 2019).

In a bleomycin-induced pulmonary fibrosis model, TRPC6-deficient mice show reduced production of collagen and an almost normal function of the respiratory system, which can be explained by the upregulation of TRPC6, increased calcium flow and localization of nuclear factor of activated T cells (NFAT) in wild-type primary murine lung fibroblasts (Hofmann et al., 2017). In addition, TRPC6 gain-of-function mutations contributed to focal segmental glomerulosclerosis (FSGS) (Szabó et al., 2015). Renal interstitial fibrosis induced by unilateral ureteral obstruction (UUO) is associated with increased expression of TGFβ1, *a*-SMA, collagen and other fibrosis-related markers and a dramatic upregulation of TRPC3/C6, which can be abrogated by genetic knockout of these channels in mice (Hofmann et al., 2017). A further study on the role of TRPC6 in dermal fibroblasts revealed the TGFβ1-mediated signaling cascade, which starts with the p38-MAPK phosphorylation and nuclear translocation of serum responsive factor and ends in the TRPC6 gene expression (Davis et al., 2012).

Inflammatory bowel diseases (IBDs) featuring chronic intestinal inflammation, which includes ulcerative colitis (UC) and Crohn’s disease (CD), can cause detrimental fibrosis. In the intestinal myofibroblast cell line, TGFβ1 intervention enhances *α*-SMA stress fiber formation, TRPC6 mRNA and protein expression and calcium influxes. Inhibition of TRPC6 by RNA interference or dominant-negative mutations effectively suppresses TGFβ1-induced calcium influxes, *a*-SMA expression, and stress fiber formation and increases ERK 1/2, Smad 2 and p38 phosphorylation and antifibrotic cytokines, such as IL-10 and IL-11 (Kurahara et al., 2015). These results indicate that the augmented calcium influxes caused by TRPC6 upregulation facilitate stress fiber formation by downregulating TGFβ1/ERK-mediated IL-10 and IL-11 production. Interestingly not all TRP channels have profibrotic roles. The activation of TRPA1 can lead to the release of inflammatory tachykinins, such as substance P and neurokinin A, and calcitonin gene related peptide (CGRP), which has been shown to be protective in the dextran sodium sulfate (DSS)-induced colitis model as well as in UC patients (López-Requena et al., 2017; Utsumi et al., 2018). The IBD activity index is significantly elevated in TRPA1 knockout compared with the wild-type mice, with a greater level of substance P, neurokinins A and other inflammatory neuropeptides, cytokines and chemokines (Kun et al., 2014). Collectively, these results confirm the anti-inflammatory role of TRPA1.

Apart from organ fibrosis, TRPs are also linked with tumor progression and fibrosis, as fibrosis commonly occurs and affects tissue rigidity (Petho et al., 2019). Overexpression of TRPC1 promotes EMT in breast cancer (Azimi et al., 2017) and TGFβ stimulation in invasive ductal breast carcinoma (Schaar et al., 2016). TRPM7 channel expression can regulate epidermal growth factor (EGF)-induced signal transducer and activator of transcription 3 (STAT3) phosphorylation and expression of the EMT marker vimentin in human breast cancer cells (Davis et al., 2014). TRPV4 can promote cell stiffness through the calcium-dependent/AKT/E-cadherin signaling axis (Lee et al., 2017), thus influencing tumor cell metastasis (Lee et al., 2016). Considering that the production of extracellular protein metalloproteinase-9 (MMP-9) is mediated by AKT signaling in cancer (Lee et al., 2017), TRPV4 may also affect tissue stiffness in this manner.

#### 3.1.2 Piezo Ion Channel

The Piezo family, including Piezo1 and Piezo2, is activated by various types of mechanical stimuli and functions as a biological pressure sensor. Piezo proteins contain 2,100–4,700 amino acids, encompassing 29–34 transmembrane segments, and do not bear sequence homology to other already known voltage-sensitive channels (Wang and Xiao, 2018). It has been proposed that Piezo proteins consist of discrete pore modules and mechanotransduction modules, which are coordinated for ion conduction, mechanical force sensing, and transduction (Ge et al., 2015; Zhao Q et al., 2016). The mechanotransduction module does not require any additional proteins to perform its mechanical sensing function (Wang and Xiao, 2018). Piezo 1/2 cationic channels are nonselective and permeable to sodium, potassium, and calcium (Murthy et al., 2017).

In zebrafish outflow tract valve morphogenesis, Piezo was found to regulate Yes-associated protein (YAP) localization and the expression of Klf-2 and ECM proteins, suggesting that Piezo channels function through the YAP1 and Klf2-Notch signaling axes (Duchemin et al., 2019). In liver sinusoidal endothelial cells, mechanical stress can be sensed via integrins, and the molecular interactions between integrins and Piezo1 then activated Piezo channels and cause them to bind to the Notch1 receptor, leading to the expression of the downstream transcription factors, Hes1 and Hey1 and finally upregualting CXCL1 production (Hilscher et al., 2019). CXCL1, as a neutrophil chemoattractant, induces sinusoidal thromboses, portal hypertension and fibrogenesis. In a recent study, increased Piezo1 expression was found in hypertrophic scars and was shown to participate in scar formation (He et al., 2021).

#### 3.1.3 Calcium Channels

The major downstream effect of ion channel activation in cellular mechanotransduction is the change in cytoplasmic calcium concentration (Malakou et al., 2018). An oscillation of the intracellular calcium level induced by mechanical stimuli has been observed in cardiomyocytes (Takahashi et al., 2019), keratinocytes (Elsholz et al., 2014), and myofibroblasts (Godbout et al., 2013), indicating that mechanical stimuli may directly regulate the fate of these cells by modulating calcium signals (Lv et al., 2015). Apart from nonspecific cation channels, such as TRP and Piezo, which have been discussed previously, voltage-gated calcium channels (Cav) (Atlas, 2014) and calcium-sensing receptors (CaSR) (Lee and Lee, 2018) also participate in the control of calcium homeostasis.

CaSR is a member of the class C GPCR. A study found that CaSR expression in zebrafish lateral-line hair cells regulates mechanotransducer-channel-mediated calcium entry (Lin et al., 2018), suggesting that CaSR is involved in mechanotransduction and could be a potential therapeutic target. In renal ischemia–reperfusion mice, a sustained influx of Orai1+ CD4 T cells with increased IL-17 expression and intracellular calcium concentration was observed. Blockade of the store-operated calcium channel Orai1 using 2APB could attenuate IL-17 + cell activation and aggravate the inflammation, fibrosis, and impaired renal function (Mehrotra et al., 2019). Another study in which Orai1 was blocked in a UUO mouse model also showed decreased expression of fibronectin, *α*-SMA, and TGFβ1 in the kidney cortex (Mai et al., 2016). In an *in vivo* experiment examining l-phenylalanine, a fibroblast suppressor targeting CaSR, a release of endoplasmic reticulum calcium stores was observed, accompanied by disruption of intracellular calcium homeostasis triggering cell apoptosis via the endoplasmic reticulum or mitochondrial pathways (Wang B et al., 2018). In animal models of fibrosis following tissue injury, poly (*p*-dioxanone-co-l-phenylalanine) reduces skin scarring and suppresses peritoneal fibrosis and postoperation adhesion (Wang B et al., 2018), making it a promising translational agent.

### 3.2 Signaling Pathways in Cellular Mechanotransduction

The activation of signaling pathways in response to mechanical cues shows significant importance in normal physiology, while its complexity presents an obstruction to potential therapeutic intervention (Tschumperlin et al., 2018). A number of transcription factors regulated by mechanical stress have been identified (Mendez and Janmey, 2012). Here, we focus on the Hippo, Wnt/*β*-catenin, and focal adhesion kinase (FAK) pathways, which are central coordinators of fibrosis-relevant mechanical responses.

#### 3.2.1 Hippo Signaling Pathway

The Hippo pathway is an evolutionarily conserved signaling cascade regulating numerous biological processes, including organ development and maintenance of tissue homeostasis. It comprises a core kinase cascade, MST1/2 and LATS1/2, and the downstream transcriptional coactivators YAP and WW domain-containing transcription regulator protein 1 (TAZ) (Dupont, 2016). In normal cells, YAP/TAZ are localized in the cytoplasm in their inactivated phosphorylated form, when activated, they localize to the nucleus and upregulate their target genes. The primary binding partners of YAP/TAZ are TEAD family transcription factors (Zhou et al., 2016). Their translocation and binding to TEAD can induce transcriptional programs that are important for cell proliferation, survival, and migration (Ma et al., 2019). The Hippo signaling pathway can be regulated by upstream mechanical signals (Totaro et al., 2018). For example, the increase in matrix stiffness leads to multiplication of the adhesive area, which promotes YAP/TAZ nuclear localization and targeted gene induction (Dupont, 2016). RhoA GTPase, which is modulated by changes in cell geometry and controls YAP/TAZ translocation by promoting actin polymerization, is believed to be the key characteristic in transducing stiffness signals (Meliambro et al., 2017). C-Jun N-terminal kinase (JNK) and its phosphorylation of LIMD1, which can directly bind to LATS1/2 to downregulate its kinase activities in increased matrix stiffness, have also been observed (Codelia et al., 2014), suggesting that the Hippo pathway, particularly the LATS1/2-mediated YAP phosphorylation, is involved in YAP regulation by matrix stiffening. These studies show that YAP/TAZ play pivotal roles in relaying physical cues to gene expression and cellular responses. However, the exact mechanism by which YAP/TAZ is regulated by various mechanical signals is not entirely understood.

The Hippo signaling pathway has been shown to contribute to the pathogenesis of fibrosis in organs, including the lung, kidney, liver and cardiac tissue, with hyperactive YAP/TAZ accumulation (Kim et al., 2019). Upon skin wounding, an increase in nuclear YAP/TAZ has been observed in the basal cell layer of the migrating epidermal tongue at the wound edge (Walko et al., 2017), and also in suprabasal cells (Elbediwy et al., 2016). It is likely that mechanical stress influences the intracellular localization of YAP/TAZ in lung fibroblasts, and YAP/TAZ increase the expression of connective tissue growth factor (CTGF, also known as cellular communication network factor 2, CCN2), Serpine one and ECM-related proteins such as collagens and fibronectin (Liu et al., 2015). In a UUO mouse model, YAP/TAZ activation led to TGFβ-induced EMT-like features in renal tubulointerstitial fibrosis (Seo et al., 2016). In liver fibrosis, TAZ-mediated Indian hedgehog gene expression plays a key role in the transition from steatosis to nonalcoholic steatohepatitis (Wang et al., 2016). In addition, the Hippo signaling pathway can regulate the renewal and regeneration of cardiomyocytes (Heallen et al., 2013), and it was found to be pathologically activated in arrhythmogenic cardiomyopathy, a myocardial disease characterized by the replacement of cardiac myocytes by fibro-adipocytes, cardiac dysfunction, and arrhythmia (Chen et al., 2014). Apart from fibrotic disorders, the Hippo pathway was also found to be associated with tumor fibrosis (Rognoni and Walko, 2019; Zheng and Pan, 2019). In mammary cancer-associated fibroblasts (CAFs), YAP activation is responsible for ECM remodeling (Calvo et al., 2013) and cytoskeletal reorganization (Foster et al., 2017). The myocardin-related transcription factor (MRTF) pathway, another mechanosensitive transcriptional regulatory network, was found to be activated in CAFs (Medjkane et al., 2009). The YAP and MRTF pathways exhibited mutual dependence and formed a positive feedback loop (Foster et al., 2017), that is governed by cytoskeleton dynamics.

#### 3.2.2 Wnt/*β*-Catenin Signaling Pathway

*β*-catenin is a component that consisting of intercellular adhesive junctions. *ß*-catenin directly binds to *α*-catenin, which mechanically links type-I classical cadherins to F-actin (Leckband and de Rooij, 2014). Isolated *ß*-catenin can stretch and refold in discrete steps, and this conformational change mechanically regulates interactions of *ß*-catenin domains with other cytosolic proteins (Valbuena et al., 2012). However, in this mechanical connection, *α*-catenin acts as an essential physical linker between the cadherin-*β*-catenin complex and the actin cytoskeleton (Desai et al., 2013; Veeraval et al., 2020), while *ß*-catenin is required only to link cadherin to *α*-catenin (Jung et al., 2019).

In the mouse model that expresses K14-ROCK:ER, actomyosin contractility, collagen density, and tissue stiffness are increased as a consequence of ROCK activation. Following the activation of ROCK, *ß*-catenin localization changes from membranous to cytoplasmic and nuclear, with an apparent increase in the overall *ß*-catenin level, and more importantly, nuclear *ß*-catenin is found in its activated form (Kümper and Marshall, 2011; Li and Wang, 2020).

In the UUO model of renal fibrosis, the expression of 19 Wnt proteins and 10 Fzd receptors has been found to be increased in renal tubular cells (Malik et al., 2020), and the accumulation of active *ß*-catenin induces fibrogenesis progression, including EMT and epithelial dedifferentiation (Zhou et al., 2013). Dickkopf1, a Wnt antagonist that binds the LRP5/6 receptor and inhibits the canonical Wnt pathway, reduces *ß*-catenin accumulation and fibrosis, downregulates collagen deposition, decreases interstitial expansion and reduces *α*-SMA production (Malik et al., 2020). Some small molecules, such as the vitamin D analog paricalcitol, have been suggested to inhibit the Wnt pathway by competing with TCF-4 (Boughanem et al., 2020).

In HBV/HCV-associated hepatocellular carcinoma, HCV upregulates the expression of *ß*-catenin and MYC, and HBV upregulates the expression of EPCAM, *ß*-catenin and MYC and activates nuclear factor κ-B (NF-κB) signaling (Arzumanyan et al., 2013). In human fibrotic liver tissue, roof plate-specific spondin protein, a Wnt pathway agonist, is overexpressed and enhances Wnt pathway activity, promoting hepatic stellate cell (HSC) activation (Yin et al., 2016).

In pulmonary fibrosis, airway damage in alveolar epithelial cells can promote canonical WNT/*β*-catenin signaling pathway activation, inducing the remodeling of interstitial fibroblasts, and the persistent remodeling finally results in pulmonary fibrosis (Knudsen et al., 2017). In pulmonary capillary endothelial cells, repeated injury can cause the suppression of CXCR7 expression and the recruitment of vascular endothelial growth factor receptor 1-expressing perivascular macrophages, which upregulates the Notch ligand Jagged1 in a Wnt/*β*-catenin-dependent manner and in turn stimulates Notch signaling to enhance fibrosis (Cao et al., 2016).

#### 3.2.3 FAK Signaling Pathway

FAK, also known as protein tyrosine kinase 2 (PTK2), is composed of an N-terminal FERM (protein 4.1, ezrin, radixin and moesin sequence homology) domain, a central kinase domain, three proline-rich regions and a C-terminal focal-adhesion targeting (FAT) domain (Murphy et al., 2020). It functions as an important mediator of integrin and growth factor signaling. FAK is recruited by the integrin-binding proteins paxillin and talin to focal contacts (Samarel, 2014), where the ECM, integrins, and the cell cytoskeleton interact. FAK can be activated by autophosphorylation (Miller et al., 2020), creating an SH2-domain-containing protein binding motif (Lagares and Kapoor, 2013) and, thereby creating a functional bipartite kinase complex (Burridge et al., 2019), which in turn further phosphorylates FAK and releases its full enzymatic activity (Miller et al., 2020). The activity of FAK is dependent on integrin-mediated cell adhesion, and it also participates in transduction pathways that are activated by growth factors (Lagares and Kapoor, 2013). Many integrin complexes are able to sense ECM stiffness and consolidate the adhesive bonds formed through vinculin-dependent reinforcement, which promotes mechanotransduction and enhances FAK activation (Sun et al., 2016).

In the human gingival fibroblast model, increased expression of FAK with type I collagen production and stress fiber formation has been observed in response to mechanical stress (Wei et al., 2020). The FAK signaling pathway is also associated with dermal fibrosis. In human scleroderma fibroblasts, increased FAK expression, induced by autocrine TGF*β* signaling, can enhance the *a*-SMA production (Van De Water et al., 2013). FAK is activated under cutaneous injury conditions, and physical loadings can potentiate this process. By inhibiting FAK, scar formation is attenuated with reduced monocyte chemoattractant protein-1 signaling and inflammatory cell recruitment, indicating that mechanical cues regulate dermal fibrosis through the inflammatory FAK-ERK-MCP-1 pathway (Wong et al., 2011).

The FAK signaling pathway is also involved in tumor induced fibrosis. In the p48-Cre; LSL-Kras(G12D); Trp53 (flox/+) (KPC) mouse model of human pancreatic ductal adenocarcinoma (PDAC), FAK inhibition can downregulate the fibrosis with a decrease in collagen deposition, fibroblasts, and *α*-SMA production, and without an acceleration of tumor progression (Jiang et al., 2016). By applying cyclic mechanical stretching to RAW264.7 murine macrophagess, the cells showed enhanced M1 polarization and tumoricidal effects with the activation of the FAK/NF-κB signaling pathway. Furthermore, while injecting the mechanical stretch-preconditioned macrophages into murine melanomas *in vivo*, a decrease in tumor cell proliferation and increase in tumor cell apoptosis has been observed by inhibiting hyperactive PI3K/AKT signaling (Shan et al., 2019). These research outcomes suggest the potential of the FAK signaling pathway as a therapeutic target in the tumor microenvironment regulation and tumorigenesis.

In pulmonary fibroblasts, FAK expression and activity are upregulated with JNK activation and profibrotic gene expression. When inhibited by the targeted siRNAs in a bleomycin-induced lung fibrosis mouse model, abrogation of fibrosis has been observed (Zhao X.-K et al., 2016). Recombinant IL-32*γ* could significantly attenuates collagen deposition and *α*-SMA production through the integrin-FAK-paxillin signaling axis in both bleomycin-induced pulmonary fibrosis mouse models (Hong et al., 2018).

Similarly, the FAK signaling pathway plays an essential role in the activation of HSCs and liver fibrosis progression. TGFβ-induced FAK activation promotes the *α*-SMA expression, while the inhibition of FAK activation blocks the *α*-SMA and collagen expression and inhibits the formation of stress fibers (Zhao et al., 2017).

In a TGFβ1-or UUO-induced renal fibrosis model, overexpression of phosphatase and tensin homolog deleted on chromosome ten (PTEN) inhibits the FAK signaling pathway. Silencing PTEN enhances fibrosis, which can be significantly reversed by the FAK inhibitor PF567721. These findings suggest that PTEN can promote renal fibrosis through the FAK/AKT signaling pathway (Du et al., 2019).

## 4 Cellular Mechanoeffecting in Fibrosis

Cellular mechanoeffecting is the final link in the response to mechanical cues, which includes the activation and transcription of specific genes, translation of microRNAs, and expression and effect of the corresponding proteins. The intracellular part is tightly controlled by epigenetic modifications, and the cytoplasm part is regulated by microRNAs. In this part, we mainly focus on epigenetic modifications and mechanosensitive microRNAs.

### 4.1 DNA Methylation

DNA can be methylated at position C5 of the pyrimidine ring of cytosine residues by DNA methyltransferases (DNMTs), and the process comprises three steps: enzyme addition of a methyl group onto cytosine, enzyme removal of the methyl group, and DNMT recognition and binding to the methyl group to eventually influence gene expression (Moore et al., 2013). A study revealed that hemodynamic force and shear stress can regulate endothelial nitric oxide synthase gene expression through posttranscriptional mechanisms (Chen and Fu, 2020). In 5-azacytidine-treated cells plated on soft matrix, a decrease in DNA methylation levels has been observed, accompanied by a decrease in histone deacetylase 1 (HDAC1) transcription, an increase in the expression of pluripotency genes and activation of the Hippo signaling pathway (Pennarossa et al., 2018), suggesting that the cells could sense matrix rigidity and react through epigenetic mechanisms.

DNA methylation is globally upregulated in pulmonary fibrosis. However, DNA hypermethylation and hypomethylation can both be observed at locus specific methylation levels (O'Reilly, 2017). Thy-1, an important regulator of cell–cell and cell–ECM interactions, is downregulated in lung fibroblasts by DNA hypermethylation, and its absence promotes the myofibroblast differentiation. Similarly, cyclooxygenase-2/prostaglandin E2, a key antifibrotic pathway inhibiting fibroblast activation and collagen deposition, is diminished with upregulated DNMT3a expression and activity in pulmonary fibrosis (Dowson and O’Reilly, 2016). Desmoplaki (DSP) is a known ECM stiffness-regulated mechanosensitive gene. In stiff matrix circumstances, a conserved region in the proximal DSP promoter becomes hypomethylated or even demethylated, resulting in the EGR1-dependent DSP overexpression, the effects of which can be blocked by CRISPR/dCas9/DNMT3A-mediated epigenetic editing (Qu et al., 2018).

### 4.2 Histone Modification and Chromatin Remodeling

Histone modification is a reversible process that indicates the covalent posttranslational modification of histone proteins, including methylation, acetylation, phosphorylation, adenylation, ubiquitination, sumoylation, and ADP ribosylation (Lyu et al., 2019). Histone acetylation is the most studied process and is controlled by histone acetyltransferases (HATs) and histone deacetylases. Hemodynamic forces, pulsatile shear and oscillatory shear can modulate the expression of HDAC to regulate anti-inflammatory and antioxidant signaling by altering the acetylation of transcription factors (Lee and Chiu, 2019). Furthermore, a study identified that mechanical stress can alter histone acetylation through actin filament-mediated sequestration, suggesting the role of the cell cytoskeleton in the nuclear-cytosolic shuttle of HDACs (Li et al., 2013).

The dysfunction of the HDACs is associated with various fibrotic diseases. Class IIa HDACs can interact with myocyte enhancer factor 2 (MEF2), decrease its expression, and attenuate myocardial hypertrophy (Zhang D et al., 2018). Class I and IIb HDACs are also involved in cardiac remodeling (Jin et al., 2017). Apart from acetylation, histone lysine demethylase (KDM) has shown regulatory effects in cardiac hypertrophy and fibrosis. In response to pressure overload, KDM3A, an H3K9me2-specific demethylase, activates Timp1 transcription to promote left ventricular hypertrophy and fibrosis. JIB-04, a pan-KDM inhibitor, suppresses pressure overload-induced hypertrophy and fibrosis (Zhang Q.-J et al., 2018).

In a bile duct ligation-induced liver fibrosis mouse model, HDACI intervention was found to effectively reduce the HSC activity and ameliorated hepatic dysfunction (Park et al., 2014). In Schistosomiasis-induced liver cirrhosis, HDACIs induce apoptosis of the larvae and adult form of schistosomula by inhibiting the NF-κB signaling pathway, while inducing the production of anti-inflammatory cytokines and reducing the number of activated macrophages (de Oliveira et al., 2017). These observations suggest a role for HDACI in dampening the inflammatory reaction, reducing hepatic injury and ameliorating hepatic fibrogenesis.

In UUO-induced renal fibrosis, all four class IIa HDAC isoforms are upregulated in renal epithelial cells. Administration of MC1568, a selective class IIa HDACI, suppresses the expression of *α*-SMA, fibronectin, and type I collagen, reduces the phosphorylation of Smad3 and NF-κB, and induces the production of ɑvβ6 integrin, suggesting that it alleviates renal fibrosis by inhibiting the TGF*β*1-induced response and promoting antifibrotic protein production (Xiong et al., 2019). Histone modifications are also found to be involved in renal fibrosis in diabetic kidney disease by promoting the expression of profibrotic factors, accelerating the accumulation of ECM and stimulating EMT progression (Sun et al., 2017).

In IPF pulmonary tissues, a significant upregulation of Class I and II HDAC activation has been reported (Korfei et al., 2015). NCC170, an HDAC8-selective inhibitor, could ameliorates TGFβ1-induced fibroblast contraction and *α*-SMA expression in normal human lung fibroblasts. Furthermore, NCC170 significantly reduces the expression of type I collagen and fibronectin in a bleomycin-induced pulmonary fibrosis mouse model (Saito et al., 2019). These studies reveal the contribution of HDACs in pulmonary fibrosis and support their potential as therapeutic targets.

### 4.3 Mechanosensitive microRNAs in the Cellular Response

**FIGURE 3:**
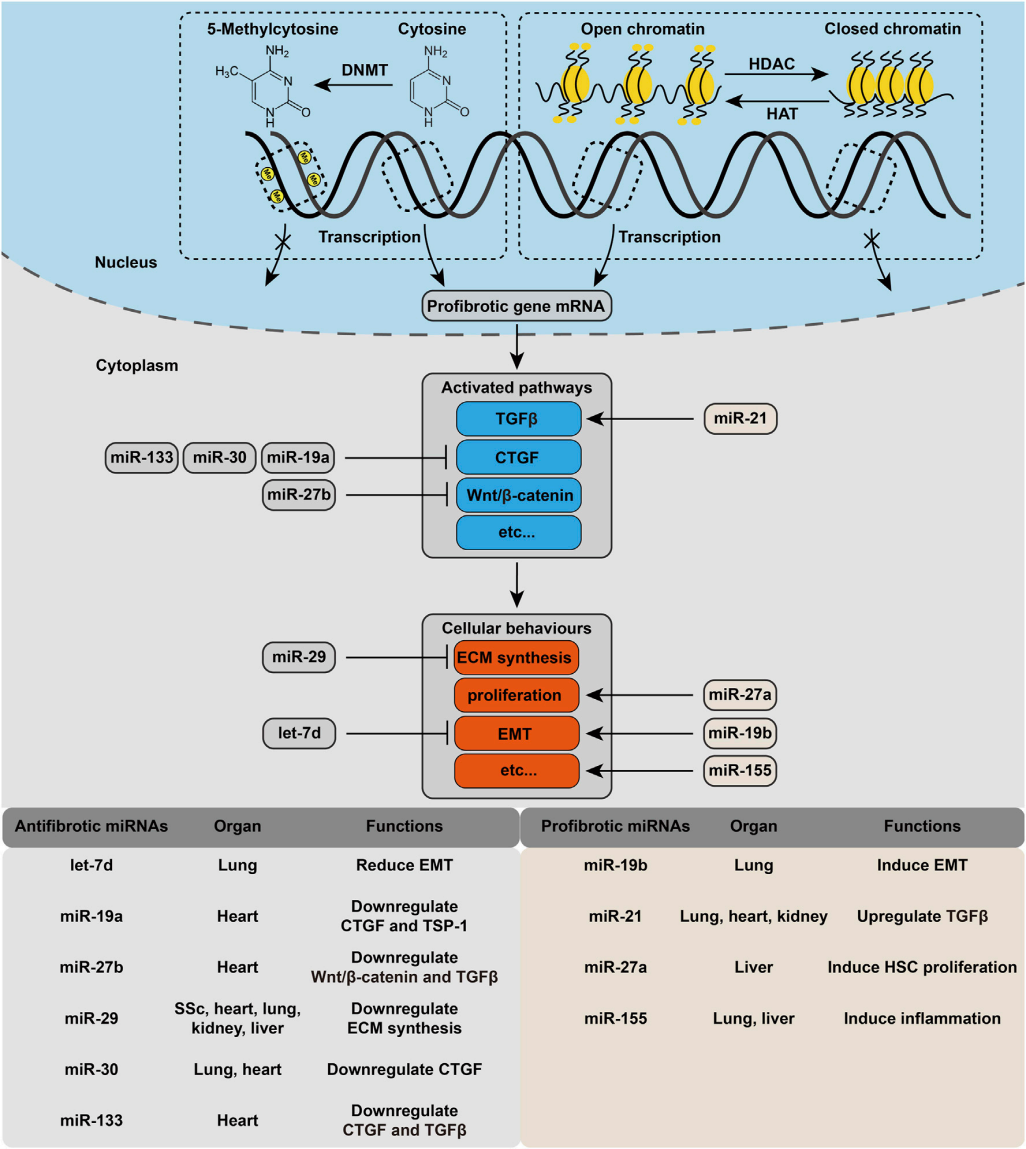
Cellular mechanoeffecting mechanism in fibrosis. Each step of the activation of profibrotic pathway is under regulation of different kind of epigenetic modification. Several mechanosensitive miRNAs are also involved in this process.

MicroRNAs (miRNAs) are a group of small noncoding RNAs with a strictly regulated biogenesis that function as negative regulators by binding to the 3′UTR of target mRNAs and degrading them. Arpproximately 50 miRNAs have been implicated in the pathogenesis of fibrotic disease (O'Reilly, 2017; Vettori et al., 2012). Figure 3 lists the mechanosensitive miRNAs involved in fibrogenesis, and the details of each miRNA are provided below.

MiR-19a/b belong to the miR-17–92 cluster. MiR-19a has been shown to regulate CTGF and thrombospondin-1 (TSP-1) with widespread ECM accumulation in a mouse model of age-related heart failure (Vettori et al., 2012), and laminar shear stress has been found to increase the expression of miR-19a (Kumar et al., 2014), suggesting its antifibrotic effect in cardiac remodeling. Interestingly, in response to mechanical stress, miR-19b downregulates PTEN in human lung epithelial cells, leading to activation of the AKT pathway and mechanical stretch-induced EMT phenotypes (Mao et al., 2017), which make it a profibrotic miRNA.

MiR-29 is the most studied direct regulator of ECM synthesis, which has been reported to be downregulated, accompanied by the consequent elevation of collagen expression in a myocardial infarction model (Ren et al., 2020), human systemic scleroderma (SSc) dermal fibroblasts and a SSc skin (Saveria Fioretto et al., 2020), salt-induced hypertensive renal fibrosis (Liu Y et al., 2010), a bleomycin-induced pulmonary fibrosis mouse model (Cushing et al., 2011), and a nonalcoholic steatohepatitis mouse model (Jiang et al., 2017). The expression of miR-29 was found to be under the control of mechanotransduction pathways, particularly in a YAP/TEAD-dependent manner. Further study verified that in IPF, mechanotransduction of ECM stiffness can negatively regulate miR-29 (Klinkhammer et al., 2018).

MiR-133 and miR-30 are two major regulators of CTGF expression in cardiac fibrosis. MiR-133 is a cardiac-specific miRNA, while miR-30 is not limited to heart tissues (Vettori et al., 2012). MiR-133 and miR-30 both mediate the expression of CTGF in a mechanical pressure overload-induced heart disease animal model, with downregulation of the two miRNA families and upregulation of CTGF *in vivo* (Tang et al., 2020). MiR-133 was further found to downregulate TGF*β*1 in a canine model of nicotine-induced AF (Yousefi et al., 2020). A recent study indicated that miR-30 can regulate osteoblast differentiation under mechanical pressure in a mechanosensitive manner (Zhang et al., 2020).

Cells can store memories of cumulative mechanical stress exposure, with YAP/TAZ acting as an intracellular mechanical regulator and potentially including genetic, epigenetic or structural changes (Yang et al., 2014). In mesenchymal cells (MSCs), miR-21 has been recognized as a long-term memory keeper of the fibrogenic program (Li et al., 2017). In mouse MSCs, ECM stiffness causes the upregulation of miR-21, which is under the control of MRTF-A nuclear translocation. After the removal of mechanical stimuli, the miR-21 level remains high for over 2 weeks. Moreover, knocking down miR-21 by the end of the stiff-priming period erases the mechanical memory and sensitizes MSCs to subsequent exposure to soft substrates (Li et al., 2017). Apart from cardiac fibrosis, miR-21 has also been confirmed to have a profibrotic role in human IPF, murine bleomycin-induced lung fibrosis (Liu G et al., 2010) and renal fibrosis (Zhong et al., 2011).

MiR-27b is significantly downregulated in AF patients, accompanied by reduced connexin 43 expression. Using an adenovirus to overexpress miR-27b-3p was observed to reduce the duration of AF, ameliorate atrial fibrosis, increase connexin 43 expression and decrease the expression of type I/III collagen, *α*-SMA, TGFβ1, Wnt3a and p-*β*-Catenin, indicating that miR-27b-3p regulates the Wnt/*β*-catenin signaling pathway by targeting Wnt3a (Lv et al., 2019). In cardiac fibrosis, miR-27b was also found to inhibit Smad-2/3 phosphorylation by targeting ALK5 (Wang Y et al., 2018). In contrast, miR-27a was found to induce lipid accumulation and proliferation of HSCs in hepatic fibrosis, showing a profibrotic effect (Ji et al., 2009).

MiR-155 is a multifunctional miRNA, and in fibrotic diseases, it mainly plays a profibrotic role. In liver fibrosis and alcohol-induced steatohepatitis, miR-155 promotes inflammation to induce fibrosis (Nazari-Jahantigh et al., 2012). In SSc, miR-155 overexpression is required for NLRP3 inflammasome-mediated collagen synthesis (Artlett et al., 2017). In silicosis mice, inhibition of miR-155-5p ameliorates macrophage and fibroblast activation and thus exerts antifibrotic effects (Chen et al., 2020), making it a promising therapeutic strategy. In atherosclerosis, miR155 has been found to regulate atherogenesis by promoting inflammatory responses (Du et al., 2014), and the expression of which can be increased by laminar shear stress, making it a mechanosnesitive miRNA (Kumar et al., 2014).

## 5 Current Clinical Trials in Fibrotic Diseases

Fibrosis is associated with diverse diseases in different organ systems. In recent years, our understanding of the complex pathogenesis continues to grow, and the advances in researches pave the way for novel clinical strategies in diseases management. However, despite the insights we had made in the cellular and molecular level, or the pathogenetic models we brought up to mimic fibrogenesis, in clinical practice there are few effective therapies and even fewer methods focusing on mechano-regulation. Thus highlighting the need for a deeper comprehension of fibrogenesis and the translation from laboratories to bedsides. Here we summarized the current or recently ended studies targeting mechano-regulation (Supplementary Table S1), as well as mechanosensitive biomarkers (Supplementary Table S2) in fibrotic diseases, and we discussed the possible strategies targeting mechano-sensing, mechano-transduction and mechano-effecting process. Hopefully the novel measures promise to stabilize the fibrotic conditions, ameliorate symptoms, improve life qualities and ultimately reverse and cure fibrosis.

### 5.1 Strategies Targeting Cellular Mechanosensing

The *α*v*β*6 integrin has been shown to be upregulated in patients with liver diseases and correlated with the stage of fibrosis (Hintermann and Christen, 2019). In mouse models of carbon tetrachloride-induced hepatic fibrosis and bleomycin-induced pulmonary fibrosis, inhibitors of *α*v integrins showed potent antifibrotic effects (Conroy et al., 2016), and similar outcomes were found in renal fibrosis (Conroy et al., 2016). In IPF patients, BG00011 and simtuzumab (Supplementary Table S1) were tested for their safety and efficacy. BG00011, a humanized monoclonal antibody against *α*v*β*6 integrin, could interfere with the cell–ECM mechanotransduction and suppress TGFβ activation. Simtuzumab was thought to disturb the collagen cross-linking to reduce tissue stiffness and tension by binding LOXL2. However, the clinical evaluation of simtuzumab was terminated due to lack of efficacy. Strategies to manipulate *α*v integrins, such as antibody blockade and small molecule inhibitors, could potentially be effective antifibrotic therapies.

### 5.2 Strategies Targeting Cellular Mechanotransduction

In CF patients, epigallocatechin gallate, tocotrienol and quercetin were found to increase the CFTR related ion channel activity to regulate disease progression. Two clinical trials were designed to evaluate their effectiveness and safety (Supplementary Table S1). Nifedipine, a calcium channel blocker, was found to prevent fibrotic changes in a bleomycin model of pulmonary fibrosis (Mukherjee et al., 2015). While nifedipine had little or no effect on lung inflammation, its protective effect might be prompted by disrupting calcium oscillation in fibroblasts.

Melatonin has inhibitory effects on the expression and activation of YAP by binding to MT1/MT2 melatonin receptors in bleomycin-induced mouse lung fibrosis models (Zhao et al., 2018). Morin increases the expression of MST1 and decreases that of YAP/TAZ in the a diethylnitrosamine-induced liver fibrosis rat model and hepatic stellate cells derived from humans (Perumal et al., 2017). And Verteporfin inhibites YAP transcriptional activity by interfering with YAP-TEAD interactions (Brodowska et al., 2014). Considering that the MRTF and YAP signaling pathways are controlled by an interlocking loop, the inhibition of one pathway could result in inhibition of the other. MRTF could be a novel therapeutic target. The MRTF inhibitors CCG-1423 and CCG-203971 were found to have antifibrotic potential in mouse skin (Haak et al., 2014), lung (Sisson et al., 2015) and vascular (Minami et al., 2012) fibrosis models. In a rabbit model of scar tissue formation after glaucoma filtration surgery, which is a validated and preclinical model of fibrosis, local administration of the MRTF-A inhibitors CCG-203971 and CCG-222740 significantly decrease fibrosis (Yu-Wai-Man et al., 2017). However, considering the central role of the Hippo pathway in organ development and regeneration, these strategies may also cause undesired harmful effects.

### 5.3 Strategies Targeting Cellular Mechanoeffecting

In a pressure overloading-induced cardiac hypertrophy mouse model, administration of the DNMT inhibitor RG108 was found to diminish the fibrotic response and downregulated a set of genes, including Atp2a2 (encodes one of the SERCA calcium-ATPases) and Adrb1 (encodes the *β*1-adrenoceptor) (Stenzig et al., 2018). This research suggested a link between DNMT inhibitor treatment and the attenuation of cardiac hypertrophy. The DNA methylation level can also be used as a biomarker for fibrosis management. In patients with nonalcoholic fatty liver disease, plasma DNA methylation of peroxisome proliferator-activated receptor γ (PPAR*γ*) has been found to be correlated with changes in hepatocellular tissue, making it possible to evaluate liver fibrosis severity in a noninvasive way (Hardy et al., 2017).

HDAC inhibitors have also been evaluated as agents for correcting pathological fibrotic remodeling. Currently, four HDACIs have been approved by the U.S. Food and Drug Administration (FDA) for clinical use in hematologic tumors (Qin et al., 2017), while HADCIs in fibrotic diseases are still being examined in preclinical studies. For example, trichostatin A, a pan HDACI, has shown benefits in multiple fibrosis models (Lan et al., 2015; Wu et al., 2017; Yoon et al., 2019), and SK-7041, a class I HDAC-selective inhibitor, ameliorates fibrotic conditions in cardiac and renal mouse models (Arise et al., 2020; Martinez-Moreno et al., 2020). Unlike other diseases in which one therapeutic target will be sufficient, fibrotic diseases sometimes require combined epigenetic therapy. The FDA-approved pan-HDACI Vorinostat shows greater benefit in combination with PI3K inhibitors in the treatment of cutaneous T-cell lymphoma (Rangwala et al., 2012). However, histones are not the only proteins that undergo acetylation, which means HDACI may affect enzyme activity via nonhistone protein acetylation (Yoon et al., 2019), thus contributing to fatal side effects (Kwon et al., 2016).

The use of miRNAs as therapeutics is now being widely applied. Using antago-miRNAs, an oligonucleotide antagonistic targeting specific miRNA and therefore blocking the binding and stopping the repression of the mRNA to increase mRNA levels *in vivo*, has been used for 15 years (Lu and Rothenberg, 2018). The efficacy of the miRNA let-7a mimic in a mouse model of bleomycin-induced dermal sclerosis has been successfully tested (Makino et al., 2013). Lentiviral, adenoviral, or adeno-associated viral vectors with built-in miRNA precursor constructs could also be potential therapeutics, but they may also induce unwanted immune responses toward the vectors.

## 6 Summary and Outlook

Taken together, mechanical homeostasis, which includes the whole process from mechanosensing to mechanotransduction and finally mechanoeffecting, is of great importance in balancing the physiological system. An injury in a tissue sensed by cells, whether in an ECM–cell or cell–cell manner, initiates the cellular response. The signals then pass to the intracellular space where they are processed. Finally, the downstream gene is activated and carries out its function. Fibrosis represents the failure to reestablish mechanical homeostasis, thereby inappropriately activating the mechanism mentioned above and eventually leading to progressive ECM deposition and tissue destruction. Recent advances in technology and the study of tissue in physiological pathology conditions, especially in tissue repair, fibrosis, and mechanoregulation mechanisms, help us to understand how mechanical environment homeostasis is maintained under different stimulations. While this review only focuses on mechanical mechanisms, biochemical mechanisms, which are also under in-depth investigation, are equally important in fibrogenesis. The universal role of fibrosis has been identified not only in fibrotic diseases but also in disorders such as fibrillation (Kottkamp, 2013), carcinogenesis, progression, and metastasis (Chandler et al., 2019), among others. Thus, the translation of these insights into fibrosis-based clinical and therapeutic interventions may lead to the exploration of novel approaches for different types of diseases.

## Glossary

AF: atrial fibrillation
CaSR: calcium-sensing receptor
CAF: cancer-associated fibroblast
Cav: voltage-gated calcium channel
CCN: cellular communication network factor
CD: Crohn’s disease
CFTR: cystic fibrosis transmembrane conductance regulator
CGRP: calcitonin gene related peptide
COX: cyclooxygenase
CTGF: connective tissue growth factor
DAG: diacylglycerol
DNMT: DNA methyltransferases
DSP: desmoplaki
DSS: dextran sodium sulphate
ECM: extracellular matrix
EGCG: epigallocatechin gallate
EGF: epidermal growth factor
EMT: epithelial-mesenchymal transition
ERK: extracellular regulated protein kinases
FAK: focal adhesion kinase
FAT: focal-adhesion targeting
FDA: Food and Drug Administration
FSGS: focal segmental glomerulosclerosis
GPCR: G protein-coupled receptor
HAT: histone acetyltransferase
HBV: hepatitis B virus
HCV: hepatitis C virus
HDAC: histone deacetylase
HSC: hepatic stellate cells
IBD: inflammatory bowel disease
IPF: idiopathic pulmonary fibrosis
KDM: lysine demethylase
LAP: latency-associated peptide
JNK: Jun N-terminal kinase
MAPK: mitogen-activated protein kinase
MEF: myocyte enhancer factor
miRNA: microRNA
MMP: metalloproteinase
MRTF: myocardin-related transcription factor
MSC: mesenchymal cell
NO: nitric oxide
Nox2: NADPH-dependent oxidase type 2
NFAT: nuclear factor of activated T cells
NF-κB: nuclear factor κ-B
PDAC: pancreatic ductal adenocarcinoma
PGE: Prostaglandin E
PI3K: phosphoinositol-3-kinase
PPAR*γ*: peroxisome proliferator-activated receptor *γ*
PTEN: phosphatase and tensin homolog deleted on chromosome ten
PTK: protein tyrosine kinase
ROS: reactive oxygen species
RGD: arginine-glycine-aspartic acid
*α*-SMA: *α*-smooth muscle actin
SSc: systemic scleroderma
STAT3: signal transducer and activator of transcription 3
TAZ: WW domain-containing transcription regulator protein
TSP: thrombospondin
TGF*β*: transforming growth factor *β*
TRP: transient receptor potential
UC: ulcerative colitis
UUO: unilateral ureteral obstruction
YAP: yes-associated protein
