# Corrigendum: Focusing on Mechanoregulation Axis in Fibrosis: Sensing, Transduction and Effecting

**DOI:** 10.3389/fmolb.2022.894660

**Published:** 2022-04-28

**Authors:** Dongsheng Wen, Ya Gao, Chiakang Ho, Li Yu, Yuguang Zhang, Guozhong Lyu, Dahai Hu, Qingfeng Li, Yifan Zhang

**Affiliations:** ^1^ Department of Plastic and Reconstructive Surgery, Shanghai Ninth People’s Hospital, Shanghai Jiao Tong University School of Medicine, Shanghai, China; ^2^ Department of Burns and Plastic Surgery, Affiliated Hospital of Jiangnan University, Wuxi, China; ^3^ Burns Centre of PLA, Department of Burns and Cutaneous Surgery, Xijing Hospital, Fourth Military Medical University, Xi’an, China

**Keywords:** fibrosis, mechanosensing, mechanotransduction, epigenetic modification, clinical trials

In the original article, we neglected to include the funders “Innovative research team of high-level local universities in Shanghai (SHSMU-ZDCX20210400)” and “Shanghai Municipal Key Clinical Specialty (shslczdzk00901)” to the authors.

The authors apologize for this error and state that this does not change the scientific conclusions of the article in any way. The original article has been updated.

