# Supplementary material for: Focusing on Mechanoregulation Axis in Fibrosis: Sensing, Transduction and Effecting
Source: Front Mol Biosci. 2022 Mar 11;9:804680. doi: 10.3389/fmolb.2022.804680 (PMC8963247; doi:10.3389/fmolb.2022.804680)
Supplement: Supplementary file 1 [file Table1.docx]

| Conditions or diseases | | Intervention | Mechanism of action | Study description | Enrollment | Phase of development | Status | Clinical trial identifier |
| --- | --- | --- | --- | --- | --- | --- | --- | --- |
| IPF | | BG00011 | Anti αvβ6 integrin antibody | Randomized,  double-blind,  placebo-controlled | 41 | Phase II | Completed  (Has results) | NCT01371305 |
| IPF | | Simtuzumab | Anti-LOX antibody | Randomized,  double-blind,  placebo-controlled | 544 | Phase II | Terminated  (Lack of efficacy) | NCT01769196 |
| Keloid | | Botulinum toxin type A | Reduce tensile forces by promoting of fibroblasts apoptosis* | Randomized,  double-blind,  placebo-controlled | 20 | Phase II | Unknown | NCT03887377 |
| Keloid | | Remlarsen | Mimics of miRNA-29 | Randomized,  double-blind,  placebo-controlled | 14 | Phase II | Completed  (Lack of efficacy) | NCT03601052 |
| Cystic fibrosis | | Epigallocatechin gallate  and/or Tocotrienol | Activator of CFTR dependent  TRP channel | Randomized,  open label | 7 | Not Applicable | Completed  (No results posted) | NCT00889434 |
| Cystic fibrosis | | Quercetin | Activator of CFTR dependent  chloride ion channel | Open label,  single group assignment | 32 | Phase II | Completed  (No results posted) | NCT01348204 |
| Primary biliary cholangitis,  liver cirrhosis | | OP-724 | β-catenin inhibitor | Open label,  dose-ranging | 12 | Phase I | Active  (Not recruiting) | NCT04047160 |
| Liver cirrhosis | | PRI-724 | β-catenin inhibitor | Open label,  dose-ranging | 34 | Phase I/IIa | Active  (Not recruiting) | NCT03620474 |
|  | *The precise mechanism of Botulinum toxin type A is not yet completely understood, but it is thought to promote apoptosis of fibroblasts and degradation of collagens, leading to reduced tensile forces. | | | | | | | |

**Table S1** Current clinical trials targeting mechanoregulation in fibrotic diseases.

| Conditions or diseases | Biomarker | Intervention | Enrollment | Status | Clinical trial identifier |
| --- | --- | --- | --- | --- | --- |
| IPF | αvβ6 integrin | [18F]FP-R01-MG-F2 PET/CT | 41 | Recruiting | NCT03183570 |
| Myocardial infarction | αvβ3 and αvβ5 integrin | Cardiac MRI scan | 30 | Completed  (No results posted) | NCT01813045 |
| Pulmonary fibrosis | αvβ3 integrin | 68Ga-NOTA-PRGD2 PET/CT | 30 | Unknown | NCT02511197 |
| Oral submucous fibrosis | E-cadherin | Biopsy tissue analysis | 185 | Completed  (No results posted) | NCT03732872 |
| IPF | β-catenin | Lung cryobiopsy analysis | 100 | Completed  (No results posted) | NCT04187079 |
| Hepatocellular carcinoma  cirrhosis | epigenetic biomarker mSEPT9 | “Epi proColon 2.0 CE” test from Epigenomics, Inc (Berlin, Germany) | 440 | Recruiting | NCT03311152 |
| Cystic fibrosis | DNA methylation levels in 187 genomic regions (CpG dinucleotides) | Spontaneous sputum | 50 | Active  (Not recruiting) | NCT02976714 |
| Cystic fibrosis | DNA methylation levels in the promoter of 14 lung disease-modifier genes | Nasal epithelial and blood cell sample analysis | 72 | Completed  (No results posted) | NCT02884622 |
| Oral submucous fibrosis | MiRNA-21 | Saliva analysis | 185 | Completed  (No results posted) | NCT03732872 |
| IPF | MiRNA-200 family | Blood samples | 450 | Recruiting | NCT03457935 |
| Hepatopulmonary syndrome in cirrhosis | MiRNA-200 and miRNA-144 | Serum analysis | 300 | Unknown | NCT03435406 |
| Radiation-induced fibrosis in breast cancer | Heat shock proteins  (HSP27, HSP70, αβ crystalline) | Skin biopsies and blood samples analysis | 20 | Completed  (No results posted) | NCT03000764 |

**Table S2** Mechanosensitive biomarkers in fibrotic disease.
