# Supplementary figures and images for: Focusing on Mechanoregulation Axis in Fibrosis: Sensing, Transduction and Effecting
Source: Front Mol Biosci. 2022 Mar 11;9:804680. doi: 10.3389/fmolb.2022.804680 (PMC8963247; doi:10.3389/fmolb.2022.804680)

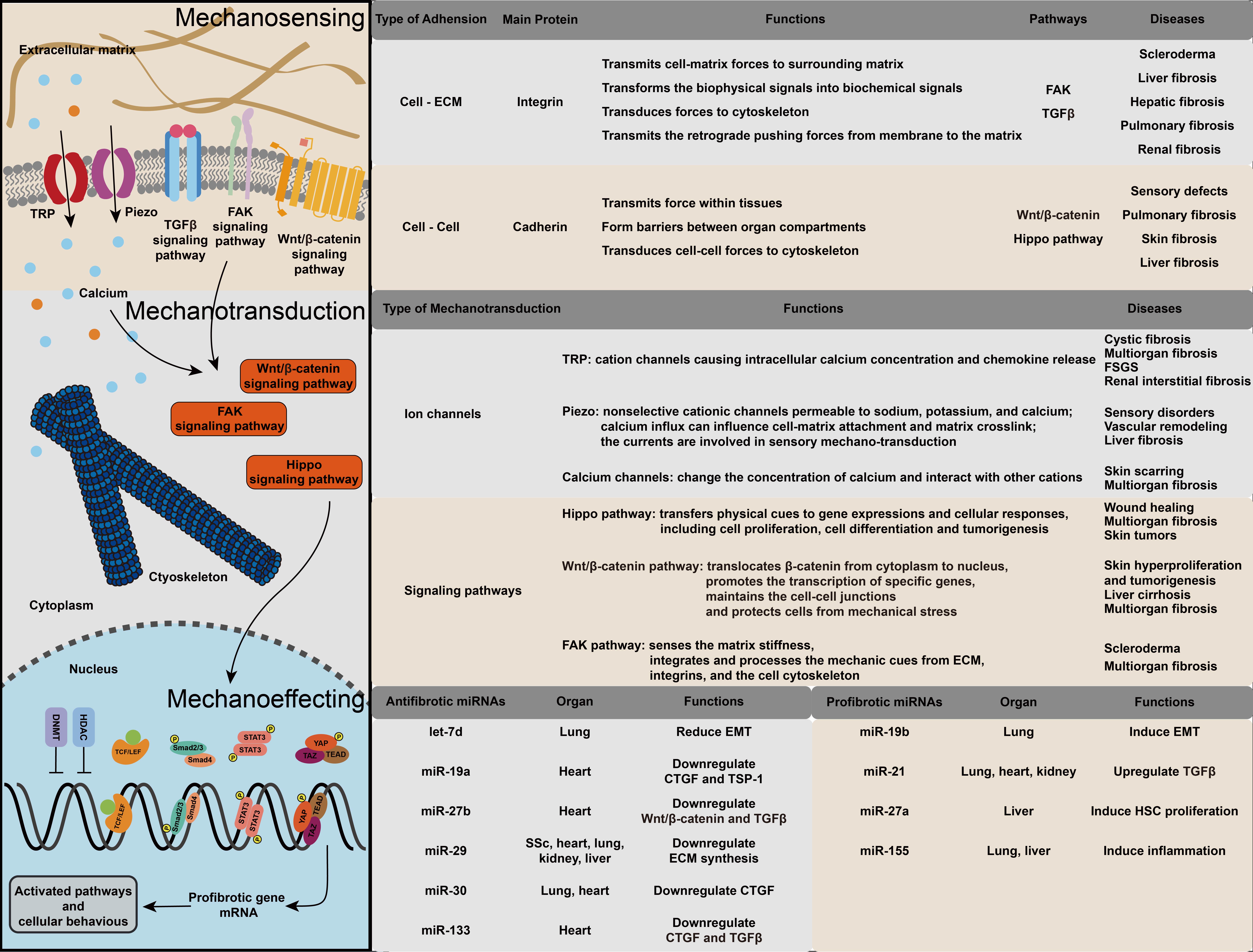

Supplement: Supplementary file 2 [file Image1.JPEG]
